# 

**Published:** 1854-04-01

**Authors:** 


### Notices to Correspondents.

WE have been compelled again to refuse several American periodicals, for which extravagant postage has been demanded.

We have been obliged to postpone the publication of several notices of British and Foreign works and periodicals until our next Number. Among these is the new edition of Dr. Conquest's "Outline of Midwifery," edited by Dr. J. M. Winn. We strongly recommend this excellent manual to the attention of our professional brethren. Its utility has been materially increased by the valuable annotations of the Editor. We should have scarcely recognised the work in its new dress. Numerous additional illustrations have been interspersed throughout the text, and several entirely new sections introduced on the subject to which it specially relates.

To give an analysis of the Editor's commentaries, which are in themselves a condensed analysis of nearly all that has been written on obstetric science during the last 16 years, would occupy too much of our space.

We scarcely know any work which contains more valuable matter in so small a compass as this work; and we strongly recommend it to the student, as one of the cheapest and best works of the kind extant. The profession ought to feel deeply indebted to Dr. Winn, for the great care and ability with which he has evidently edited this valuable manual.

